# Epigenetic profiling demarcates molecular subtypes of muscle-invasive bladder cancer

**DOI:** 10.1038/s41598-020-67850-5

**Published:** 2020-07-02

**Authors:** K. E. van der Vos, D. J. Vis, E. Nevedomskaya, Y. Kim, W. Choi, D. McConkey, L. F. A. Wessels, B. W. G. van Rhijn, W. Zwart, M. S. van der Heijden

**Affiliations:** 1 Division of Molecular Carcinogenesis, The Netherlands Cancer Institute, Amsterdam, The Netherlands; 2 Division of Oncogenomics, The Netherlands Cancer Institute, Amsterdam, The Netherlands; 3 Present Address: Department of Pathology, Cancer Center Amsterdam, Amsterdam UMC, Vrije Universiteit Amsterdam, Amsterdam, The Netherlands; 4 Johns Hopkins Greenberg Bladder Cancer Institute, Brady Urological Institute, Johns Hopkins University, Baltimore, MD USA; 5 Department of Surgical Oncology (Urology), The Netherlands Cancer Institute, Antoni Van Leeuwenhoek Hospital, Amsterdam, The Netherlands; 6 Laboratory of Chemical Biology and Institute for Complex Molecular Systems, Department of Biomedical Engineering, Eindhoven University of Technology, Eindhoven, The Netherlands; 7 Oncode Institute, The Netherlands Cancer Institute, Amsterdam, The Netherlands; 8 Faculty of EEMCS, Delft University of Technology, Delft, The Netherlands

**Keywords:** Histone post-translational modifications, Epigenetics, Bladder cancer

## Abstract

Muscle-invasive bladder cancer (MIBC) is a heterogeneous disease that often recurs despite aggressive treatment with neoadjuvant chemotherapy and (radical) cystectomy. Basal and luminal molecular subtypes have been identified that are linked to clinical characteristics and have differential sensitivities to chemotherapy. While it has been suggested that epigenetic mechanisms play a role in defining these subtypes, a thorough understanding of the biological mechanisms is lacking. This report details the first genome-wide analysis of histone methylation patterns of human primary bladder tumours by chromatin immunoprecipitations and next-generation sequencing (ChIP-seq). We profiled multiple histone marks: H3K27me3, a marker for repressed genes, and H3K4me1 and H3K4me3, which are indicators of active enhancers and active promoters. Integrated analysis of ChIP-seq data and RNA sequencing revealed that H3K4 mono-methylation demarcates MIBC subtypes, while no association was found for the other two histone modifications in relation to basal and luminal subtypes. Additionally, we identified differentially methylated H3K4me1 peaks in basal and luminal tumour samples, suggesting that active enhancers play a role in defining subtypes. Our study is the first analysis of histone modifications in primary bladder cancer tissue and provides an important resource for the bladder cancer community.

## Introduction

Muscle-invasive bladder cancer (MIBC) is an aggressive disease with a 50% mortality rate at 5 years. Radical cystectomy with cisplatin-based neoadjuvant chemotherapy is the current standard of care. Overall, neoadjuvant chemotherapy has only led to a small increase in overall survival. More recently, immune checkpoint inhibition is showing promising results in a subpopulation of patients^1–4^. Still, response rates remain low, emphasizing the challenges in bladder cancer treatment and a need for predictive biomarkers.

Genome-wide sequencing by The Cancer Genome Atlas (TCGA) has revealed that MIBC is a heterogeneous disease with a high mutational load^5,6^. Subtype stratification by RNA expression profiling can potentially help to identify the therapeutic options for individuals. Several studies describe molecular subtypes in MIBC, and several classifications have been proposed^7–11^. Recently, a collaborative effort combined transcriptome profiles from multiple cohorts and produced a consensus scheme with six molecular subtypes: luminal papillary, luminal nonspecified, luminal unstable, stroma-rich, basal/squamous, and neuroendocrine-like^12^. These subtypes are characterized by differences in mutational profile, differentiation markers, infiltrating stromal and immune cells, and clinical outcome. Importantly, these molecular subtypes appear to have differential sensitivities to neoadjuvant chemotherapy (NAC)^10,13^ and checkpoint inhibitors^14,15^, though prospective validation is still needed. Luminal subtypes have the best overall survival with and without NAC. In contrast, basal tumours associate with metastasis and poor prognosis, but they appear to benefit most from neoadjuvant chemotherapy^16^.

The luminal subtypes express markers of terminal urothelial differentiation, such as uroplakins. The basal/squamous and neuroendocrine-like subtypes show distinct expression signatures^12^. The understanding of the underlying biological mechanisms defining the molecular bladder cancer subtypes has yet to develop. Using gene expression data to find upstream regulators of the molecular subtypes, Choi et al. found that luminal MIBC exhibit peroxisome proliferator activator receptor (PPAR) pathway activation and high expression levels of PPARG^10^. Also, Eriksson et al. showed that molecular subtypes differ in the expression of urothelial differentiation programs involving PPARG/RXRA, FOXA1, GATA3, and anterior HOXA and HOXB genes^17^.

Interestingly, of all TCGA cancer types, mutations in chromatin-modifying genes were most common in urothelial cancer and encompassed histone methyltransferases and demethylases^5^. However, the extent and localization of chromatin modifications in primary bladder tumours as well as their functional consequences, remain uncharted territory. To better understand the epigenetics of bladder cancer subtypes, we assessed multiple histone methylation marks in chemotherapy-naïve primary urothelial cancer samples. We used chromatin immunoprecipitation followed by sequencing (ChIP-seq) to pinpoint genomic regions with histone methylation marks in subgroups of patients. We mapped histone 3-lysine 27 trimethylation (H3K27me3), a marker for repressed genes, and histone 3-lysine 4-mono- and tri-methylation, which are indicators of active enhancers and active promoters respectively. Here we provide a detailed overview of three histone methylation marks that gives insight into the epigenetic differences between MIBC subtypes. Also, this unique data-set of genome-wide ChIP-seq data of histone methylation marks in primary bladder tumours will serve as a valuable new resource for the bladder cancer community.

## Results

### ChIP-seq analysis of bladder tumour samples identifies chromatin regions with specific histone methylation patterns

**Figure 1:**
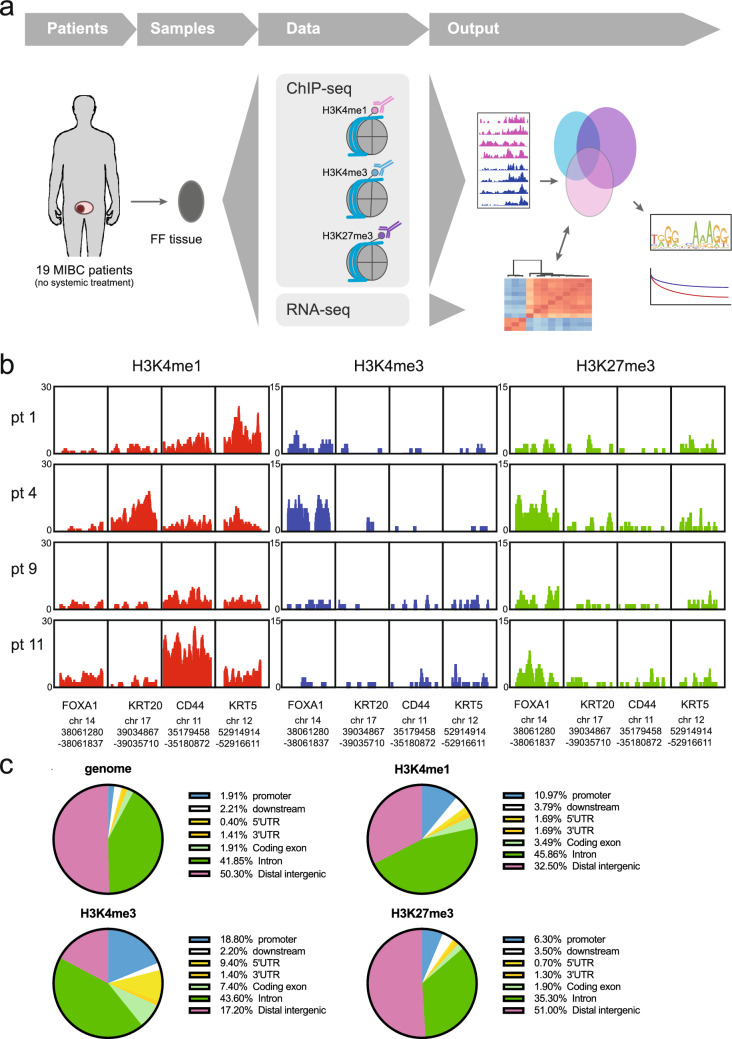
Characterization of ChIP-seq data. (**a**) Schematic outline of the study design. (**b**) Genome snapshots for H3K4me1 (green), H3K4me3 (blue), and H3K27me3 (pink), ChIP-seq are shown at four example loci in four patients. Genomic coordinates are indicated above. The y-axis shows read counts as indicated. (**c**) Genomic distribution of consensus peaks from H3K4me1, H3K4me3 and H3K27me3 chromatin immunoprecipitations across genomic features.

To better understand the epigenetics of bladder cancer subtypes, we probed the chromatin state of 19 tumours that were surgically resected (Fig. 1a). We selected fresh-frozen tumour samples from chemotherapy-naïve patients with muscle-invasive bladder cancer that underwent a cystectomy and divided these between a discovery (Table 1, n = 12) and validation (Table 2, n = 7) cohort. To investigate whether pathological features were associated with genome-wide changes in methylation marks at enhancers and promoters, we performed ChIP-seq analyses using antibodies directed against multiple histone modifications. We analysed the mono-methylation of lysine 4 on histone 3 (H3K4me1; active enhancers), tri-methylation of lysine 4 on histone 3 (H3K4me3; active promoters) and the repressive mark, tri-methylation lysine 27 of histone 3 (H3K27me3). After performing chromatin immunoprecipitation and DNA sequencing of the separate precipitates, reads were mapped to the reference human genome, and peaks were called. First, we visually inspected the ChIP-seq profiles for the histone methylation marks of four genes that mark different subtypes of MIBC: the luminal markers *FOXA1* and *KRT20* as well as the basal markers *CD44* and *KRT5*^10^. We observed clear differences between patients (Fig. 1b). Shown in Fig. 1b are the typical peaks observed for H3K4me1, H3K4me3, and H3K27me3. For downstream analyses, we focussed on reproducible peaks, that is, peaks that were present in at least three tumour samples (Figure S1). Next, we examined the genomic distribution of the consensus peaks for all three methylation marks. As expected, H3K27me3 peaks were mainly found in intergenic regions, while H3K4me1 and H3K4me3 consensus peaks showed enrichment for promoters (Fig. 1c). These results are consistent with the current knowledge of histone methylation patterns and show that primary bladder cancer tissue can be processed for ChIP-seq, producing high-quality data of genome-wide histone marks.

**Table 1:** Clinical characteristics MIBC patients—discovery cohort.

Patient	Gender	Age	Disease stage	Progression (0: no, 1: yes)	Days till progression
1	M	45	pT4bN2Mx	1	133
2	M	51	pT4N0Mx R1	1	314
3	F	74	pT3bN0Mx	0	
4	M	62	pT3bNxMx	0	
5	M	60	pT4aN0Mx	0	
6	F	63	pT3aN2M1	1	193
7	F	61	pT3N0Mx	1	146
8	M	76	pT3bN0Mx	0	
9	M	76	pT3bG3N0Mx	0	
10	M	65	pT3N0Mx	0	
11	M	62	pT3bN0Mx	0	
12	M	72	pT3aN3Mx	1	104

**Table 2:** Clinical characteristics MIBC patients—validation cohort.

Patient	Gender	Age	Disease stage	Progression (0: no, 1: yes)	Days till progression
13	M	57	pT4bN2Mx	1	245
15	F	70	pT3N1Mx	1	421
16	M	56	pT3N0Mx	1	90
17	F	55	pT4bN2M1	1	22
18	F	84	pT4aN0Mx	0	
19	M	49	pT3N0Mx	1	76
21	M	58	pT4N2Mx	1	65

### H3K4 mono-methylation patterns are associated with bladder cancer subtypes

**Figure 2:**
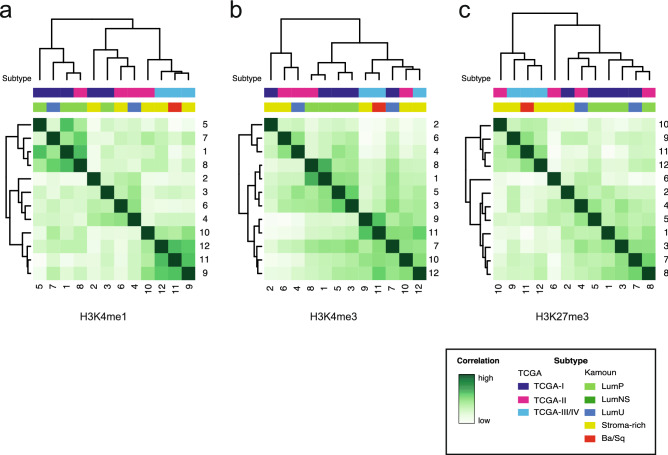
Genome-wide distribution of histone 3 methylation. Unsupervised hierarchical clustering of genome-wide consensus peaks for H3K4me1 (**a**), H3K4me3 (**b**) and H3K27me3 (**c**). Each sample is annotated for molecular subtype. Shown are correlation heatmaps based on read count.

We hypothesize that distinct histone modification profiles reflect the luminal and basal molecular subtypes. Therefore hierarchical clustering of each histone methylation mark was performed to analyse the correlation between the tumour samples based on their epigenetic status on a global scale. The grouping of H3K4me1 consensus peaks revealed the presence of three clusters (Fig. 2a). Hierarchical clustering analysis was also performed for H3K4me3 and H3K27me3, showing respectively 3 and 2 groups (and one outlier sample) (Fig. 2b,c). To gain insights into tumour characteristics, RNA from these tumours was sequenced, and molecular subtypes were assigned using the TCGA 2014 classification that consists of four subclasses: TCGA-I–IV^6^. A cross-validated multinomial regression model was trained on TCGA data and used to annotate the TCGA subtype of our tumours (Fig. S2). Our analysis was able to discriminate between the two luminal TCGA subtypes but indicated that we could not separate the TCGA-III and TCGA-IV subtypes with sufficient confidence. Therefore, we continued with an mRNA subtype classification that consists of the two luminal subtypes (TCGA-I, TCGA-II) and one basal dominated subtype, TCGA III + IV. In our discovery cohort, we identified nine luminal and three basal tumours, while our validation cohort contained four luminal and three basal tumours. Recently, a consensus molecular subtype system for MIBC has been published^12^. We have applied this method to our data and observe good overall concordance. This analysis also supports our observation that it is challenging to subdivide the basal dominated group as the separation scores are very similar, and the separation levels are low (Table S1). To investigate the biological differences between luminal and basal tumours, we compared these molecular subtypes with histone methylation clusters. Interestingly, we observed that one of the H3K4me1 clusters contained only basal tumours (Fig. 2a), suggesting that (in)activation of enhancers may drive differences between MIBC subtypes (Fig. S3). Unsupervised hierarchical clustering of H3K4me3 profiles revealed three distinct clusters that were not corresponding to the MIBC subtypes TCGA 2014 I, II, or III + IV (Fig. 2b). Similarly, the H3K27me3 profiles also did not associate with these TCGA subtypes (Fig. 2c), suggesting that the enhancer regions might define subtype differentiation independent of promoter methylation. Clustering of histone methylation profiles did not correlate with prognosis.

**Figure 3:**
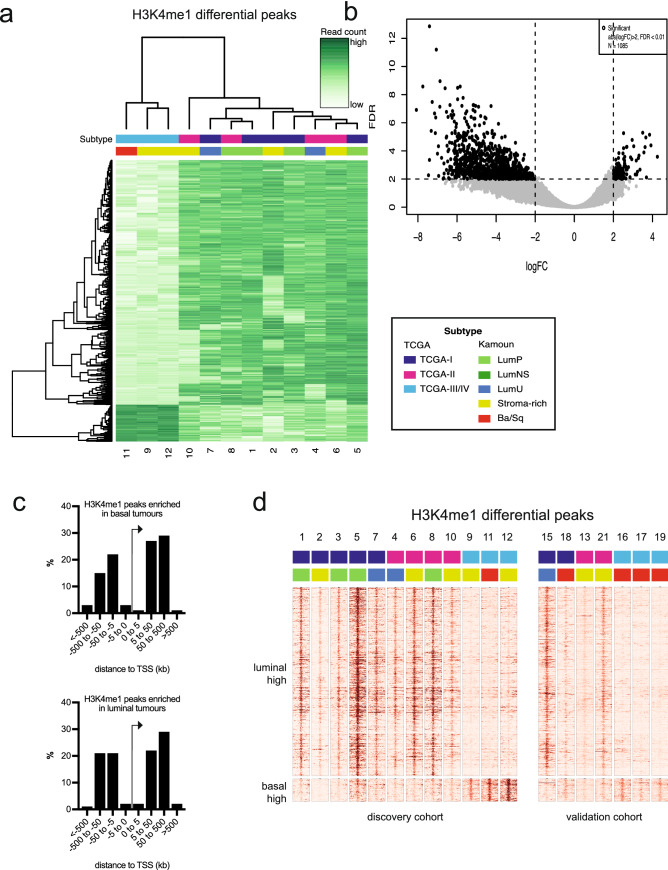
H3K4me1 defines basal subtype MIBC. (**a**) Samples were grouped based on basal and luminal subtype, and H3K4me1 peaks that show significantly differential read counts were identified. The correlation heatmap shows H3K4me1 differential peaks in the discovery cohort. (**b**) Volcano plot showing the log fold change of all H3K4me1 differential peaks in the basal subtype samples compared to the luminal tumours. Significant peaks are in bold. (**c**) Distance from TSS to peaks that are enriched in basal tumours and peaks that are enriched in luminal tumours. (**d**) Heatmaps visualizing raw read count intensity of H3K4me1 at differential binding sites in the discovery cohort and the validation cohort. Each sample is annotated for subtype.

To further investigate the epigenetic status of the basal tumours, we focussed on H3K4me1 consensus peaks that showed differential read counts between basal (TCGA III + IV) and luminal (TCGA I + II) subtypes. We identified 1,085 differential H3K4me1 consensus peaks, of which 89% were methylated in luminal tumours (Fig. 3a, b). In contrast, only 115 H3K4me1 methylation peaks exhibited higher read counts in basal tumours compared to the luminal tumours. The majority of the differential H3K4me1 peaks we identified were lying within 500 Kb of the transcription start sites (Fig. 3c). To gain more understanding of the biological processes regulated by these epigenetic marks, we performed gene ontology analysis of the genes that are near the identified peaks. This analysis revealed enrichment for differentiation processes, such as gland development and pattern specification processes, in the luminal subtype compared to the basal subtype (Tables S2, S3). These results suggest that the modulation of enhancer activity influences the maintenance of the phenotypic characteristics of the basal and luminal subtypes in MIBC.

To confirm our findings, we performed ChIP-seq analyses on a second (validation) set of MIBC tumour samples (Table 2). Read counts for the 1,085 H3K4me1 peaks that were differentially methylated between the basal and luminal subtypes were compared between our discovery and our validation cohorts. As shown in Fig. 3d, the H3K4me1 consensus peaks that we had identified to be specifically occupied by H3K4me1 in basal or in luminal bladder tumours showed similar H3K4 mono-methylation patterns in our validation cohort. These results confirm that enhancer activity associates with basal and luminal MIBC subtypes.

### H3K4me1 designated enhancers are enriched for PPARG binding motifs and contain a subset of poised enhancers

**Figure 4:**
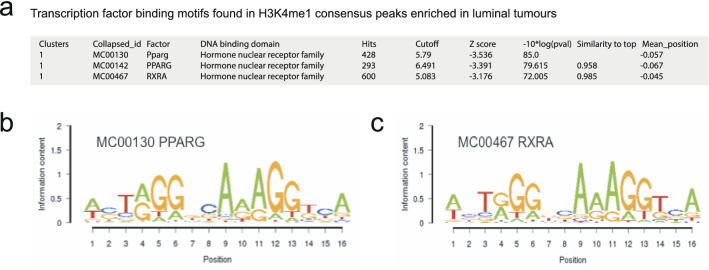
Functional analysis of H3K4me1-marked enhancers. (**a**) Differential H3K4me1 consensus peaks were analysed for enrichment of transcription factor binding motifs. Shown are the motifs that are enriched in the luminal tumours. (**b**) The PPARG consensus motif identified in the motif search. (**c**) The RXRA consensus motif identified in the motif search.

We queried the differential H3K4me1 consensus peaks for transcription factor binding motifs using the Cistrome database^18^. Interestingly, the consensus peaks that were specifically H3K4me1-positive in the luminal tumours were enriched for Peroxisome proliferator-activated receptor gamma (PPAR-γ or PPARG) and Retinoid X Receptor Alpha (RXRA) binding motifs (Fig. 4a–c). In contrast, the H3K4me1 peaks in basal tumours did not show enrichment for a specific transcription factor. PPAR-γ has been described to be involved in the differentiation of normal urothelium, and high expression of this transcription factor is associated with the MIBC luminal subtype. The identification of potential binding sites of this differentiation marker in enhancers specific for luminal tumours confirms the previously published relevance of PPAR-γ signalling in subtype stratification^10,17^.

**Figure 5:**
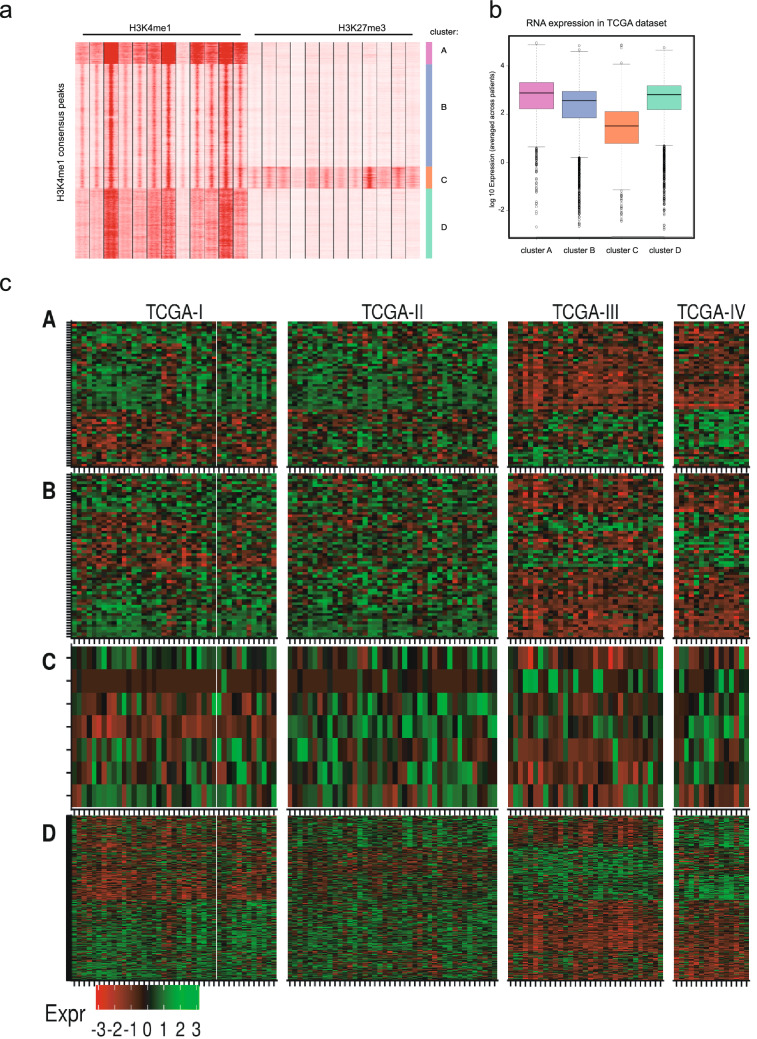
Analysis of multiple classes of enhancers. (**a**) Cluster analysis of H3K4me1 and H3K27me3 consensus peaks. (**b**) Expression of genes in the proximity of different regulatory clusters using TCGA expression data. (**c**) Heatmap of differentially expressed genes across molecular subtypes for the enhancer clusters A (super-enhancers), B (active enhancers), C (poised enhancers) and D (active enhancers).

It has been shown previously that the integration of multiple histone methylation marks can identify various classes of enhancers with distinct biological functions, including poised enhancers that showed both H3K4me1 and H3K27me3 marks and were linked to low gene expression levels^19^. We took all H3K4me1 consensus peaks that we identified in our bladder cohort and defined the H3K27me3 status of each region. Next, we performed cluster analysis on these regions. The purpose of this analysis was to identify different classes of enhancer/promoter regions, including putative super-enhancers (high H3K4me1), active enhancers (intermediate H3K4me1), and closed or poised enhancers (high H3K4me1 and high H3K27me3). As shown in Fig. 5a, the integrated analysis yielded four subclasses of enhancers; cluster A containing putative super-enhancers, clusters B and D containing putative active enhancers, and cluster C containing putative poised enhancers. To check whether the identified poised enhancers were indeed associated with genes expressed at a lower level, we performed analyses on the TCGA-bladder cancer RNA expression data. As expected, the genes located close to the enhancers co-occupied by both H3K4me1 and H3K27me3 were expressed at lower levels compared to the other enhancer clusters, indicating that they behave as poised enhancers (Fig. 5b).

Next, we performed ingenuity pathway analysis of genes proximal to enhancer regions for each cluster and determined transcription factor binding sites in proximity to these enhancer regions, stratified by cluster. This analysis showed the strongest hits to be in cluster C, the poised enhancers (Tables S4−S5). Finally, we determined for each cluster of enhancers how gene expression (associated with these enhancers) was distributed across MIBC subtypes in the TCGA 2014 data-set (Fig. 5c). For each enhancer cluster, genes proximal to differentially bound peaks are shown if their expression varied across TCGA subtypes (ANOVA, *p* < 0.01). Differentially expressed genes aligned mainly with the basal/luminal axis.

Cumulatively, these data indicate that histone methylation patterns are associated with molecular subtypes and transcriptional pathways relevant to bladder cancer biology. They confirm that epigenetic regulation plays a central role in bladder tumour subtypes. Our data may serve as a valuable resource for future studies, to provide a deeper understanding of the epigenetic regulation of bladder cancer biology and the identification of prognostic biomarkers.

## Discussion

This report details the first comprehensive analysis of histone methylation patterns in primary tumour tissue from patients with muscle-invasive bladder cancer. We successfully inventoried genome-wide histone methylation peaks for H3K4me1, H3K4me3, and H3K27me3. The results show that informative chromatin states were present in fresh frozen primary tumour samples, and ChIP-seq captured the genome-wide occurrence of histone marks in 19 tumours. Integration of the multiple histone methylation patterns with RNA-seq data revealed that H3K4 mono-methylation patterns were associated with the bladder cancer molecular subtypes. We identified differentially methylated H3K4me1 regions in basal and luminal tumour samples, and in-depth analyses of these differential peaks provided insights into the biological pathways involved in maintaining the gene expression profiles associated with the bladder cancer subtypes. As H3K4me1 is mainly found in enhancer regions of the chromatin, these data indicate that enhancers play an important role in defining subtypes. The H3K4me1 peaks that were enriched in the luminal tumours displayed enrichment for PPAR-gamma and RXRA binding motifs. This finding is consistent with previous work, which demonstrated that PPARG plays a vital role in driving the luminal subtype^10,17,20,21^.

While our understanding of the role of enhancers in tumorigenesis is still limited, multiple studies indicate that enhancer usage is changed in cancer cells favouring the expression of growth-associated genes^22^. Identification of active enhancers in multiple cancer types based on their bidirectional transcription revealed the presence of 4,102 active enhancers in bladder cancer^23^. A fraction of these enhancers were significantly prognostic, and a considerable number of enhancers were associated with clinically actionable genes, further stressing their biological relevance^23^. MIBC needs prognostic and predictive biomarkers that could guide clinical decision making. While our epigenetic analyses revealed an association between H3K4me1-marked enhancers and MIBC subtypes, we were unable to directly link the chromatin states to clinical outcomes, which is at least partially due to the small size (n = 12 in test cohort) of our patient cohort. Our integrated analyses of multiple methylation marks provided an opportunity to evaluate the different types of enhancers that play roles in defining muscle-invasive bladder cancer subtypes.

In conclusion, we here provide the first detailed chromatin methylation maps of MIBC. This study extends the current understanding of bladder cancer subtypes by identifying epigenetic mechanisms involved in gene expression. Enhancer regions, defined by mono-methylation of H3K4, were associated with the basal and luminal subtypes. While our data show a clear association between histone methylation patterns and basal and luminal phenotypes, further work will be required to elucidate how these epigenetic markers can be exploited for clinical decision-making.

## Methods

### Patients and samples

We selected chemotherapy-naive patients that underwent radical cystectomy in the AVL between 1998 and 2007. All samples were histologically diagnosed as muscle-invasive urothelial carcinoma. Fresh frozen tumour samples were selected for chromatin immunoprecipitations and RNA sequencing. The material was cut in 30-micron sections for ChIP-seq or 10-micron sections. The clinical data were obtained from the NKI-AVL Genitourinary Clinical Database. This study was approved by the NKI Translational Research Board under registration number CFMPB226. All procedures performed in this study were following the national legislation and institutional guidelines.

### ChIP-seq

Chromatin immunoprecipitations (ChIP) were performed as described previously^21^. For each ChIP, 5 µg of antibody was conjugated with 50 µl Protein A magnetic beads. The antibodies used were H3K4me1, (ab8895, Abcam), H3K4me3 (ab8580, Abcam), and H3K27me3 (39155, Active Motif). Immunoprecipitated DNA was processed for library preparation (Part# 0801-0303, KAPA Biosystems kit). Libraries were sequenced using an Illumina Hiseq2500 genome analyzer (65 bp, single-end), and aligned to hg19 using BWA (v0.5.10).

### Peak calling

Raw sequence data was aligned to hg19 using BWA (v0.5.10), followed by selecting reads with mapping quality of > 20. MACS and DFilter were used to call peaks with the following settings: (1) MACS2 broad peak calling setting with a cutoff of 0.1 ("–broad-cutoff 0.1″) and (2) Dfilter kernels of non-zero mean ('-nonzero'), bin size of 100 ("-bs = 100″) and kernel size of 30 ("-ks = 30″). Peaks identified with MACS and DFilter were used. Consensus peaks are defined as the peaks identified in at least 3 samples for each factor. Identification of differential sites and clustering analysis is done using DiffBind. Motif analysis was performed using the Galaxy Cistrome SeqPos motif tool with default settings^18^.

### RNA-seq

Samples were processed with TruSeq RNA library prep kit v2 (Illumina) and sequenced in a HiSeq 2,500 (Illumina). Sequenced reads were aligned to the human genome (hg19), and gene expression values were quantified using RSEM, which is the quantification used by TCGA. The bladder cancer TCGA gene-expression data was downloaded from SAGE synapse (syn2319855).

### Assignment of MIBC subtypes

A multinomial elastic-net model was trained on data using cluster assignment labels from the TCGA 2014 data-set^6^. Due to the relatively small size of clusters three and four, and their apparent similarity, these two clusters were merged. The tenfold cross-validated model was trained on the TCGA data and used for predicting the cluster assignment of the local samples that were ChIP’ed.

## Supplementary information

Supplementary file1

## Data Availability

All ChIP-seq and RNA-seq data generated in this study are deposited in the Gene Expression Omnibus (GEO) database under the accession numbers [will be submitted upon acceptance].
