# Supplementary material for: Epigenetic profiling demarcates molecular subtypes of muscle-invasive bladder cancer
Source: Sci Rep. 2020 Jul 2;10:10952. doi: 10.1038/s41598-020-67850-5 (PMC7331601; doi:10.1038/s41598-020-67850-5)
Supplement: Supplementary file 1 — Supplementary file1 [file 41598_2020_67850_MOESM1_ESM.pdf]

Epigenetic profiling demarcates molecular subtypes of muscle-invasive bladder cancer.

KE van der Vos[1#], DJ Vis[1#], E Nevedomskaya[1,2], Y Kim [1,2,3], W Choi[4], D McConkey[4], LFA Wessels[1,7,8], BWG van Rhijn[5], W Zwart[2,6,7], MS van der Heijden[1*]

[1] Division of Molecular Carcinogenesis, The Netherlands Cancer Institute, Amsterdam, The Netherlands.

[2] Division of Oncogenomics, The Netherlands Cancer Institute, Amsterdam, The Netherlands.

[3] Current Affiliation: Department of Pathology, Amsterdam UMC, Vrije Universiteit Amsterdam, Cancer Center Amsterdam, The Netherlands.

[4] Johns Hopkins Greenberg Bladder Cancer Institute, Brady Urological Institute, Johns Hopkins University, Baltimore, MD, USA.

[5] Department of Surgical Oncology (Urology), The Netherlands Cancer Institute - Antoni van Leeuwenhoek Hospital, Amsterdam, the Netherlands.

[6] Laboratory of Chemical Biology and Institute for Complex Molecular Systems, Department of Biomedical Engineering, Eindhoven University of Technology, Eindhoven, Netherlands.

[7] Oncode Institute, The Netherlands Cancer Institute, Amsterdam, The Netherlands.

[8] Faculty of EEMCS, Delft University of Technology, Delft, The Netherlands

[#]These authors contributed equally to this work

[*]Corresponding author

Supplementary information

Figure S1. Overlap of identified methylation peaks.

The overlap of identified peaks between samples of the discovery cohort for (**a**) H3K4me1, (**b**) H3K4me3 and (**c**) H3K27me3.

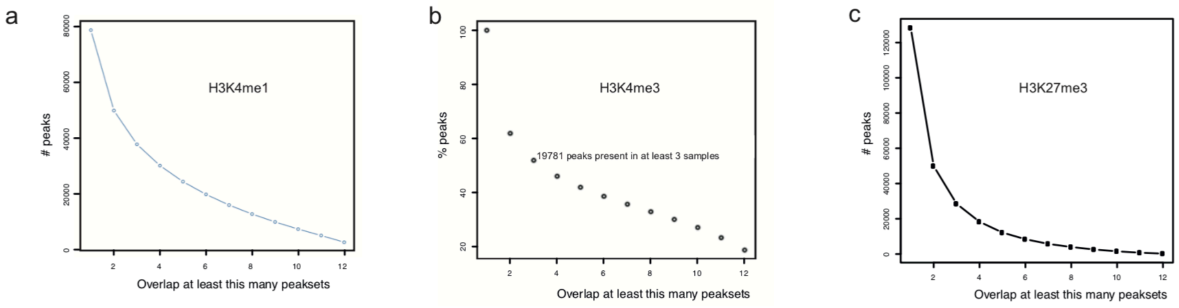

Figure S2. Subtype analysis identifies 3 TCGA-2014 subtypes of MIBC

(**a**) RNA-seq data from all patients was compared to TCGA data. Subtype analysis was performed using the TCGA classifier. For each patient, the percentage of overlap with the TCGA subtypes was plotted.

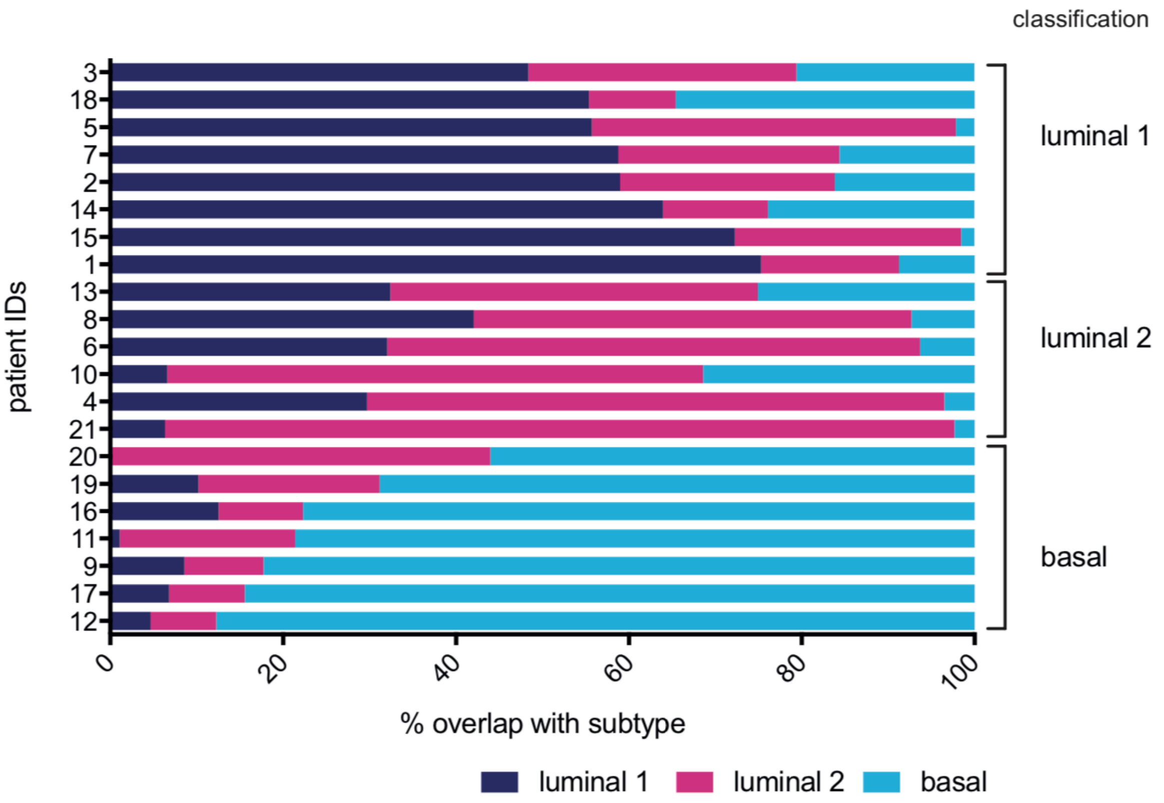

(**b**) Kaplan-Meier plot for progression-free survival of the patients used in the discovery and validation cohorts. Patients were stratified by luminal (TCGA-2014 I+II) vs basal (III+IV) subtype. The number of patients at risk at each time point is indicated.

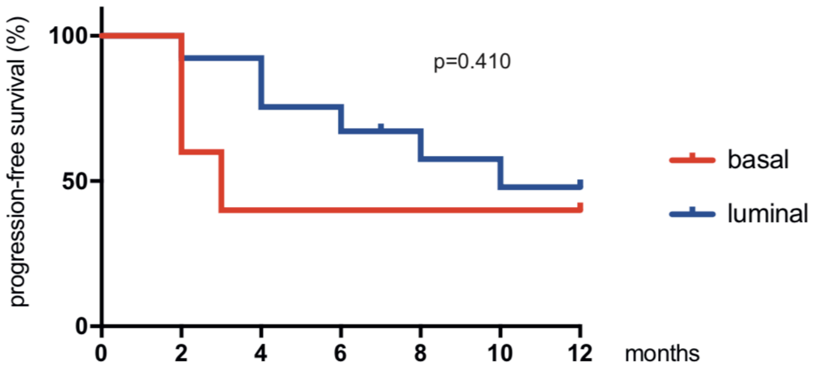

patients at risk:

	0	2	4	6	8	10	12
basal	5	5	3	3	3	3	2
luminal	13	13	11	9	7	6	5

Figure S3. Examples of differential H3K4me1 consensus peaks.

Shown are four examples of H3K4me1 consensus peaks that show differential binding of H3K4me1 between basal and luminal subtypes. Genomic coordinates and tag count are indicated.

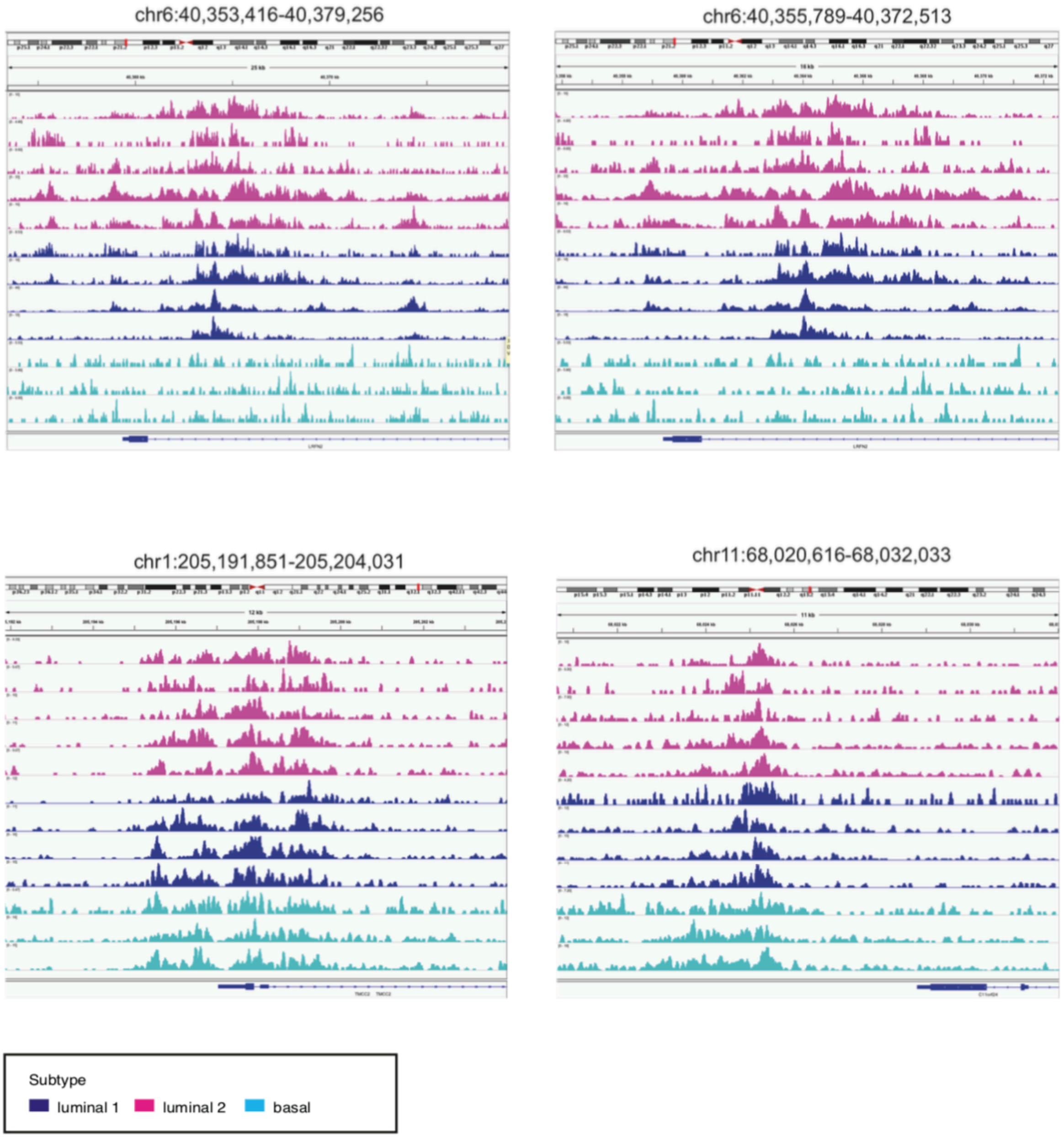

Supplemental Table S1: Kamoun subtype designation, showing separation scores for each tumor.

Patient #	consensusClass	separationLevel	LumP	LumNS	LumU	Stroma-rich	Ba/Sq	NE-like	Vos et al
1	LumP	0,51	0,38	0,35	0,36	0,33	0,26	0,18	**TCGA-I**
2	Stroma-rich	0,24	0,26	0,30	0,32	0,33	0,23	0,18	**TCGA-I**
3	LumP	0,96	0,41	0,35	0,34	0,35	0,32	0,14	**TCGA-I**
5	LumP	0,54	0,35	0,32	0,33	0,30	0,22	0,15	**TCGA-I**
7	LumU	0,39	0,38	0,41	0,42	0,40	0,30	0,22	**TCGA-I**
15	LumU	0,19	0,48	0,48	0,48	0,43	0,31	0,22	**TCGA-I**
18	Ba/Sq	0,54	0,44	0,39	0,38	0,42	0,47	0,20	**TCGA-I**
21	Stroma-rich	0,64	0,44	0,47	0,48	0,51	0,46	0,27	**TCGA-I**
4	LumU	0,10	0,33	0,36	0,37	0,37	0,25	0,22	**TCGA-II**
6	Stroma-rich	0,95	0,42	0,42	0,42	0,43	0,38	0,23	**TCGA-II**
8	LumP	0,64	0,48	0,46	0,46	0,44	0,39	0,19	**TCGA-II**
10	Stroma-rich	0,65	0,37	0,42	0,42	0,47	0,38	0,25	**TCGA-II**
13	Stroma-rich	0,15	0,42	0,46	0,44	0,46	0,37	0,21	**TCGA-II**
9	Stroma-rich	0,10	0,28	0,28	0,29	0,31	0,31	0,17	**TCGA-III/IV**
11	Ba/Sq	0,36	0,33	0,34	0,33	0,46	0,53	0,27	**TCGA-III/IV**
12	Stroma-rich	0,02	0,27	0,27	0,28	0,35	0,35	0,21	**TCGA-III/IV**
16	Ba/Sq	0,49	0,29	0,28	0,28	0,38	0,48	0,20	**TCGA-III/IV**
17	Ba/Sq	0,17	0,22	0,22	0,23	0,30	0,32	0,21	**TCGA-III/IV**
19	Ba/Sq	0,17	0,33	0,34	0,33	0,42	0,44	0,24	**TCGA-III/IV**

Table S2: Gene ontology analysis of the genes that are closest to the peaks enriched in luminal subtype tumours.

Term Name	Hyper Rank	Hyper FDR Q-Val	Hyper Fold Enrichment	Hyper Observed Gene Hits	Hyper Total Genes	Hyper Gene Set Coverage
gland development	11	0,001	2,686989	29	266	0,040
cardiac septum development	24	0,003	4,770227	12	62	0,016
mammary gland duct morphogenesis	27	0,004	6,161544	9	36	0,012
gland morphogenesis	35	0,006	3,554737	15	104	0,020
mammary gland morphogenesis	38	0,007	4,929235	10	50	0,014
mammary gland epithelium development	40	0,008	4,444392	11	61	0,015
branching morphogenesis of an epithelial tube	48	0,008	2,957541	18	150	0,025
cardiac septum morphogenesis	62	0,009	5,041263	9	44	0,012
pattern specification process	63	0,010	2,034472	35	424	0,048
outflow tract septum morphogenesis	68	0,010	10,26924	5	12	0,007
morphogenesis of a branching epithelium	78	0,012	2,691249	19	174	0,026
morphogenesis of a branching structure	91	0,018	2,572952	19	182	0,026
outflow tract morphogenesis	100	0,020	4,349325	9	51	0,012
odontogenesis	96	0,020	3,236366	13	99	0,018
mammary gland formation	113	0,027	10,95386	4	9	0,005
ventricular septum development	114	0,027	4,585335	8	43	0,011
branching involved in mammary gland duct morphogenesis	118	0,028	6,161544	6	24	0,008
signal release	126	0,032	2,738464	15	135	0,020
limb development	137	0,038	2,577378	16	153	0,022
mammary gland development	147	0,046	2,716901	14	127	0,019

Table S3. Gene ontology analysis of the genes that are closest to the peaks enriched in basal subtype tumours.

Term Name	Hyper Rank	Hyper FDR Q-Val	Hyper Fold Enrichment	Hyper Observed Gene Hits	Hyper Total Genes	Hyper Gene Set Coverage
regulation of cell communication	3	0,0010	2,505	33	2285	0,317
intracellular signal transduction	1	0,0013	3,119	26	1446	0,250
regulation of signaling	2	0,0015	2,509	33	2282	0,317
regulation of catalytic activity	5	0,0025	2,700	27	1735	0,260
phosphate-containing compound metabolic process	8	0,0030	2,488	29	2022	0,279
phosphorus metabolic process	10	0,0037	2,435	29	2066	0,279
regulation of molecular function	12	0,0045	2,390	29	2105	0,279
regulation of MAPK cascade	11	0,0048	4,584	13	492	0,125
regulation of phosphorylation	13	0,0049	3,336	18	936	0,173
phosphorylation	15	0,0067	3,226	18	968	0,173
death	23	0,0105	2,800	20	1239	0,192
regulation of intracellular protein kinase cascade	22	0,0110	3,497	15	744	0,144
cell death	21	0,0111	2,807	20	1236	0,192
regulation of heterotypic cell-cell adhesion	19	0,0119	52,041	3	10	0,029
regulation of response to stress	37	0,0209	3,158	15	824	0,144
apoptotic process	40	0,0233	2,836	17	1040	0,163
monocyte aggregation	44	0,0233	115,647	2	3	0,019
programmed cell death	45	0,0243	2,798	17	1054	0,163
positive regulation of heterotypic cell-cell adhesion	55	0,0372	86,736	2	4	0,019
negative regulation of apoptotic signaling pathway	59	0,0393	9,130	5	95	0,048

Supplemental Table S4: Ingenuity analysis of the canoncial pathways by enhancer cluster. Values represent the -log10 of the pvalue.

**Ingenuity Canonical Pathways**	**A**	**B**	**C**	**D**
Basal Cell Carcinoma Signaling			12,2	
Axonal Guidance Signaling	3,64		9,68	
Transcriptional Regulatory Network in Embryonic Stem Cells			8,77	
Cellular Effects of Sildenafil (Viagra)			7,43	
Human Embryonic Stem Cell Pluripotency			6,8	
Wnt/β-catenin Signaling			5,15	
Corticotropin Releasing Hormone Signaling			4,8	
PCP pathway			4,6	
G-Protein Coupled Receptor Signaling			4,23	
GABA Receptor Signaling			4,2	
Role of NANOG in Mammalian Embryonic Stem Cell Pluripotency			4,19	
Role of Osteoblasts, Osteoclasts and Chondrocytes in Rheumatoid Arthritis			3,74	
Role of Wnt/GSK-3β Signaling in the Pathogenesis of Influenza			3,59	
Neuropathic Pain Signaling In Dorsal Horn Neurons			3,55	
Glutamate Receptor Signaling			3,51	
Gαi Signaling			3,49	
14-3-3-mediated Signaling	3,96			
Actin Nucleation by ARP-WASP Complex	3,57			
AMPK Signaling				3,88
Aryl Hydrocarbon Receptor Signaling				3,77
CCR3 Signaling in Eosinophils	4,21			
Cholecystokinin/Gastrin-mediated Signaling	5,33			
CXCR4 Signaling	4,25			
ErbB Signaling	3,39			
Fc Epsilon RI Signaling	3,76			
Germ Cell-Sertoli Cell Junction Signaling	5,34			
Glioblastoma Multiforme Signaling	4,7			
GNRH Signaling	3,17			
HER-2 Signaling in Breast Cancer	5,24			
HIPPO signaling				3,78
Huntington's Disease Signaling	3,74			
ILK Signaling	3,05			
Insulin Receptor Signaling				3,65
Integrin Signaling	6,49			3,92
Macropinocytosis Signaling	4,17			
Molecular Mechanisms of Cancer	3,85	5,16		
NGF Signaling	3,62			
Non-Small Cell Lung Cancer Signaling	3,1			
Paxillin Signaling	3,12			
Phospholipase C Signaling	5,9			
PI3K/AKT Signaling				3,2
PTEN Signaling				3,77

RAR Activation		4,09
Regulation of Cellular Mechanics by Calpain Protease	3,2	
Semaphorin Signaling in Neurons	3,04	
Sertoli Cell-Sertoli Cell Junction Signaling	4,11	
Sperm Motility	3,04	
STAT3 Pathway	3,21	
TGF-β Signaling	3,22	
Thrombin Signaling	4,84	
Unfolded protein response	3,93	
Virus Entry via Endocytic Pathways	3,45	

Table S5: Ingenuity analysis of the upstream transcription factors by enhancer cluster. Values represent the -log10 of the pvalue.

Upstream Regulator	A	B	C	D
SOX2			12,5452	
POU5F1			11,4634	
NANOG			7,09259	
MYOCD			5,23582	
HOXB3			5,11862	
ZNF217			4,17522	
SBDS			3,98297	
HSPA9	2,10679		3,96658	
SP1			3,63451	
estrogen receptor	4,83268		3,48945	
MED12			3,42946	
NRXN1			3,40561	
KRT14			3,34679	
SPDEF			3,07779	
HOXB1			3,07058	
NEUROD1			3,07058	
SOX5			3,07058	
CXCR4			2,77211	
GATA4			2,74958	
CDX2			2,64397	
FOXL2			2,61439	
CHD7			2,50031	
CRYAB			2,50031	
FZD8			2,50031	
RPL11			2,50031	
Pka			2,46725	
CTNNB1			2,46218	
ITGAV			2,44977	
PITX2			2,3851	
HAND2			2,27003	
TBX5			2,27003	
REST			2,25727	
ERG		3,0511	2,19928	
E2F5			2,16368	
UBE3A			2,16368	
ALK			2,1343	
BMPR1A			2,1343	
IFNL2			2,1343	
IL6ST			2,1343	
ZBTB7B			2,1343	
MYOC			2,08302	
SMAD3			2,07109	

BPTF			2,04672	
KAT2A			2,04672	
LMO4			2,04672	
NF1			2,04672	
RBBP7			2,04672	
TRRAP			2,04672	
UGDH			2,04672	
WASL			2,04672	
TP53	7,08355	3,90658		4,47756
IgG	6,49894			
mir-122	5,87615			3,56543
SYVN1	5,81531			
ERBB2	5,60555			2,35262
TGFB1	5,60555			
KMT2D	5,29671			
MAPK9	4,15739			
MED1	4,10073			
MGEA5	3,2668	3,8729		2,58336
VPRBP	3,25259			2,07988
NR3C1	3,20971			2,74958
TP73	3,18709			
WISP2	3,05404			
FGFR1	3,04191			
EHF	3,03763			
DPY30	2,98716			
TP63	2,88941			
RXRA	2,86328			
Interferon alpha	2,84466			
ZEB1	2,71897			
SNAI2	2,5817			
PRDM5	2,55284			
ADAM12	2,54212			
GSTP1	2,54212			
TRIM41	2,54212			
MYCN	2,5376			
TNF	2,46597			3,18575
FOXA1	2,39469			
VHL	2,37572			
FH	2,35458			
SKI	2,31785			
SND1	2,25571			
Growth hormone	2,25337			
NME1	2,18177			
IL6	2,15181			
ESR1	2,14026			
ETV5	2,13549			

SUZ12	2,12033	2,10568	
PRKAR1A	2,10679		
MTOR	2,07058		2,51856
A2M	2,05552		
RB1CC1	2,00174		
SLC9A3R1	2,00174		
NUPR1		4,24413	
RNF31		2,84771	
E2F4		2,67985	
mir-486		2,58838	
miR-486-5p (and other miRNAs w/seed CCUGUAC)		2,58838	
NMNAT1		2,50307	
PARP1		2,2668	
E2F1		2,25414	2,31695
Lh			5,64782
FSH			4,95078
CXCL12			4,79048
mir-182			3,86967
PI3K (family)			3,52288
HIF1A			3,33161
HSF1			3,16558
GSK3A			3,15864
mir-96			3,15864
PTH			3,09691
Pkc(s)			2,95861
APP			2,91721
GSK3B			2,91364
KLRC4-KLRK1/KLRK1			2,87615
LDL			2,74232
CDK8			2,67985
EPAS1			2,6073
P38 MAPK			2,59176
ATF4			2,51999
Igm			2,51856
ECSIT			2,46092
PRKAC			2,46092
SPRY2			2,46092
IRF2			2,43533
CANX			2,32239
NOV			2,32239
PRNP			2,32239
STAT			2,32239
TBPL1			2,32239
mir-183			2,31876
FABP2			2,31695

MBD1	2,24872
STAT3	2,18243
Gsk3	2,15552
NDUFA13	2,04576
SIRT6	2,04576
MNT	2,03105
